# Engineering biology and climate change mitigation: Policy considerations

**DOI:** 10.1038/s41467-024-46865-w

**Published:** 2024-03-26

**Authors:** Jonathan Symons, Thomas A. Dixon, Jacqueline Dalziell, Natalie Curach, Ian T. Paulsen, Anthony Wiskich, Isak S. Pretorius

**Affiliations:** 1 Australian Research Council (ARC) Centre of Excellence in Synthetic Biology, Macquarie University, Sydney, NSW 2109 Australia; 2 School of History and Philosophy of Science, University of Sydney, Sydney, NSW Australia; 3 HydGene Renewables, Sydney, NSW Australia; 4 Commonwealth Scientific and Industrial Research Organisation (CSIRO), Brisbane, QLD Australia

**Keywords:** Climate-change policy, Synthetic biology, Climate-change mitigation

## Abstract

Engineering biology (EngBio) is a dynamic field that uses gene editing, synthesis, assembly, and engineering to design new or modified biological systems. EngBio applications could make a significant contribution to achieving net zero greenhouse gas emissions. Yet, policy support will be needed if EngBio is to fulfil its climate mitigation potential. What form should such policies take, and what EngBio applications should they target? This paper reviews EngBio’s potential climate contributions to assist policymakers shape regulations and target resources and, in so doing, to facilitate democratic deliberation on desirable futures.

## Abstract (web summary)

Engineering biology is a dynamic field that uses gene editing, synthesis, assembly, and engineering to design new or modified biological systems. Here the authors discuss the policy considerations and interventions needed to support a role for engineering biology in climate change mitigation.

## Introduction

Analysis of national commitments made under the Paris Agreement suggests that, without some radical discontinuity, limiting global warming to no more than 1.5 °C will prove unattainable^1^. In this article, we examine one potential source of discontinuity and the policies that might enable it: the possibility that advances in engineering biology (EngBio) might transform the economic and political feasibility of reaching net zero greenhouse gas emissions. EngBio, which is largely synonymous with ‘synthetic biology’, includes research and development into the technical themes of (i) gene editing, synthesis and assembly; (ii) biomolecule, pathway and circuit engineering; (iii) host and consortia engineering; and (iv) data integration, modelling, and automation. These themes have application and impact on the sectors of (i) industrial biotechnology; (ii) health and medicine; (iii) food and agriculture; (iv) environmental biotechnology; and (v) energy^2^. This paper has a broad focus because the most pressing challenge is for national and international climate policy-making bodies, such as the Intergovernmental Panel on Climate Change (IPCC), to begin to characterise EngBio’s emissions reduction potential across multiple technology pathways.

This article identifies factors that will shape the feasibility of deployment of EngBio applications that contribute to mitigating climate change, outlines types of policy support appropriate for EngBio applications at different stages of development, and summarises key developments in EngBio and the associated ‘bioeconomy’ that might advance mitigation. The bioeconomy, which we define as economic activity related to the life sciences research enterprise, draws on advances in the life sciences, biotechnology, engineering, computing, and information sciences and typically involves the use of biomass in the creation of energy, and intermediate and final products^3^.

EngBio’s mitigation potential is not always well understood by the climate policy community. For example, although IPCC scenarios include some EngBio applications (e.g. production of synthetic animal proteins), the IPCC has not yet systematically assessed EngBio’s potential. By contrast, EngBio practitioners engaged in ‘horizon-scan’ assessments routinely emphasise the field’s vast promise regarding climate adaptation, mitigation, and creation of a ‘circular economy’^4^. There is also a considerable basic science research effort focused on climate-linked applications—from microbial solar fuels to direct air greenhouse gas capture. Multiple reports by individual governments have also emphasised the scale of this potential mitigation contribution^5,6^. We recommend that future IPCC reports should include a systematic review of EngBio’s potential to ensure that a rigorous assessment of the field’s potential informs global and national climate policy deliberations. The next logical step is to ask: what policy interventions can ensure that advances in basic science impact global emissions trajectories?

Supporting novel EngBio applications through to deployment can be a high-risk, slow, and costly task because it involves industrial scaling of biological processes. Private investment is concentrated in the medical, pharmaceutical, chemical, and agricultural sectors because venture capital will only fund developments that promise privately appropriable benefits (appropriability refers to the extent to which the social benefits of an EngBio application can be captured as commercial benefits)^2^. In comparison, governments have traditionally supported development in areas that are perceived to align with national priorities, such as defence and health security. Operation Warp Speed, which rapidly applied EngBio to developing mRNA Covid-19 vaccines in the United States, is a prominent example^7^. Some EngBio applications promise both commercially appropriable benefits and side-benefits in the form of reduced emissions; this set of applications will likely attract private finance. For instance, private corporations have already commercialised synthetic dairy proteins (replacing methane-producing cows) and nitrogen-fixing microbes (replacing fossil fuel-derived fertilisers)^8,9^. EngBio can also contribute to climate change adaptation, for instance, by developing crops that can withstand climatic extremes. However, this paper focuses on mitigation (including sequestration of atmospheric carbon) rather than on applications of EngBio that will assist in adaptation to climate change.

EngBio applications, whose primary benefit is the positive externality of avoided greenhouse gas (GHG) emissions, do not bring privately appropriable economic benefits. Exclusively mitigation-focused applications will not attract significant private financing unless a policy intervention makes them profitable. Taxing carbon at a level that reflects the full social cost of GHG emissions is one measure that, if it were politically feasible, could be anticipated to increase private support for developing EngBio applications. However, in the absence of effective global carbon pricing, policies focused on commercialising and deploying EngBio applications — akin to those that nurtured wind and solar energy through decades of development^10^ — will be needed if EngBio is to fulfil its mitigation potential.

## Review of policy support for EngBio climate applications

Public sector support for EngBio’s climate-linked applications is underdeveloped^11^. Recently, several mitigation-focused agencies have recognised and begun to address the gap. One significant example is the US government’s *Advanced Research Projects Agency–Energy* (ARPA-E). In 2021, ARPA-E established an *ECOSynBio* programme which 'aims to promote the use of advanced synthetic biology tools to engineer novel biomass conversion platforms and systems'^12^. ARPA-E appoints technically accomplished Programme Managers on 5-year contracts, allowing them to identify and fund high-risk, high-reward, use-inspired research. While ARPA-E’s design is modelled on the successes of the Defense Advanced Research Projects Agency (DARPA), it is unclear whether ARPA-E SynBio projects will receive the kind of government procurement support that has been central to DARPA’s success. Moreover, ARPA-E is focused only on energy-sector applications and so will not address the full range of EngBio applications. Also, the 16 projects within the ARPA-E ECOSynBio programme account for only about 2% of ARPA-E’s research effort.

Global governance mechanisms promoting low-carbon innovation are also slowly incorporating EngBio. A key example is *Mission Innovation*—an international initiative that seeks to coordinate and enhance national investments in low-carbon innovation. In 2016, Mission Innovation established a 'converting sunlight' challenge that at first did not canvas a role for EngBio. However, an expert working group established to advance the Mission identified the potential to utilise photosynthetic microorganisms with engineered metabolic pathways^13^. In April 2022, Mission Innovation launched an 'Integrated Biorefineries Mission' to utilise EngBio to unlock CO_2_ emissions reduction in the transport, chemicals, and materials sectors^14^. Mission Innovation focuses on basic research rather than deployment; its increasing focus on EngBio illustrates growing awareness of the sector’s mitigation potential rather than an answer to the policy challenge of how to best advance the deployment of EngBio-enabled mitigation.

In September 2022, the Biden Administration issued an 'Executive Order on Advancing Biotechnology and Biomanufacturing Innovation for a Sustainable, Safe, and Secure American Bioeconomy' with the stated goal of identifying 'innovative solutions in health, climate change, energy, food security, agriculture, supply chain resilience, and national and economic security'^15^. This executive order prompted the US Department of Energy to develop very bold EngBio-linked mitigation goals (e.g. to 'utilise >60 million metric tons of exhaust gas CO_2_ suitable for conversion to fuels and products' by 2043)^5^. Such goals are among the priorities to be advanced by a new 'Office of Critical and Emerging Technology', which was established in December 2023^16^. Recent US legislation has also provided potential financing (e.g. tax credits in the Inflation Reduction Act that might be accessed by synthetic fuel producers)^17^ and general support for EngBio research—e.g. creation of a 'National Engineering Biology Research and Development Initiative' by the Chips and Science Act (2022). Similar patterns are apparent internationally^6^. Clearly the prominence of EngBio in national security, economic and environmental planning is rising. Yet, the realisation of EngBio’s climate mitigation potential will require an additional step: policy interventions focused directly on bridging the gap between basic science and deployment of applications that are primarily aimed at reducing greenhouse gas emissions.

### Policy considerations

If public sector support for mitigation-focused EngBio is to advance beyond basic science, policymakers must decide which applications warrant public support and what form this support should take. Table 1 maps a wide range of attributes of EngBio that are policy-relevant regarding climate change mitigation. Identifying which technologies warrant support requires both an assessment of *technical potential* and also an assessment of *political feasibility* that considers factors such as resource requirements, environmental impacts and social acceptance^18^. Social acceptance changes over time and across cultures and may also change as public understanding of the gravity of the climate crisis deepens^19^. Where an EngBio application appears technologically and politically feasible, decisions about the *type of policy support* needed to bring an application to technological maturity and deployment will be influenced by factors that include technological readiness, appropriability, and regulatory barriers to deployment (lock-out).

**Table 1:** Policy-relevant attributes of EngBio climate mitigation applications

	Type	A: Replace fossil fuels	B: Reduce emissions from production processes	C: Substitute emissions-intensive products	D: Sequester or mitigate in environment
	**Examples**	Biofuel for jet fuel, diesel, petrol.	Ethanol from CO_2_ waste streams; carbon capture and sequestration.	Synthetic meat and milk.	Carbon in crop roots.
**Global effects linked to social acceptance**	Risks/Benefits	Benefits: EngBio promises second-gen biofuels produced from ag waste streams. Risks: First-generation biofuels typically compete w. ag land use, leading to deforestation and higher food prices.	Benefits: Reduces geopolitical risks from transitioning from fossil fuels. Risks: GHGs from upstream emissions; verification risk; risk of legitimation of ongoing fossil fuel if emissions reductions do not eventuate.	Benefits: Potential to displace ag land use, leading to lower deforestation and lower food prices. Risks: social license concerns; risks to rural livelihoods and associated heightening of inequality.	Benefits: potential low-cost form of negative emissions with side-benefits for agricultural productivity. Risks: CO_2_ sequestration lacks permanence; social license concerns and potential ecological risks from engineered species
	Land/Resource impact	Value shift from oil extraction to cropland.	Preserves value of fossil fuel reserves.	Value shift from livestock land to cropland and fermentation.	Favours cropland, areas with greater soil carbon opportunity.
**Technological potential**	Appropriability	High.	Low-Medium.	High.	Low.
	Technology readiness (price comparison)	High for first gen biofuels; low for production from cellulosic biomass.	Medium.	Medium (not yet economically competitive)	Low
**Deployment context**	Infrastructure readiness	High - allows decarbonisation of existing fossil fuel equipment.	Medium, may potentially extend life of existing industry plants but requires additional infrastructure.	Medium, requires scale-up of biofermentation plants.	Development and social license for deployment of engineered crops/organisms.
	Risk of ‘lock-out’	Support from ag industry but risk of opposition from the fossil fuel industry	Support from existing industries and fossil fuel producers.	High possibility of opposition from established producers.	Incentives needed to reward established industries for participation
	Impacts	Benefit for fossil fuel importers.	Benefit countries with comparative advantage in industrial process, or fossil extraction	Expand productive areas; benefit for food current importers.	Benefit for crop producers.
**Policy mode**		Basic science & commercialisation; Public support per output.	Basic science & commercialisation; Public support per CO_2_.	Address Lock out. Public support per CO_2._	Basic science; commercialisation; knowledge sharing; democratic deliberation.

Key factors influencing political feasibility include the following:

Feedstock and land-use implications: EngBio applications may utilise captured carbon or agricultural waste (e.g. waste phytomass from corn or wheat production) as a feedstock, or they may require valuable feedstocks that have other uses (e.g. sugars, oils) or that compete with other land uses (e.g., forests). EngBio applications competing with other valued land and resource uses may be technically and politically feasible at small scales; however, large-scale deployment will likely encounter political resistance.

Displacement of existing industries: where EngBio processes directly displace existing industries (e.g. petrochemical products and animal agriculture), the politics of deployment will be influenced by the political power of vested interests (companies, unions, etc.) as well as social acceptability. Policy design should thus be attentive to patterns of deployment and transition that can accommodate these groups’ interests, or to assembling coalitions capable of overcoming socially undesirable uses of political power.

Industrial versus ecosystem deployment: public attitudes and perceptions of risk toward applications of EngBio within industrial processes (e.g. use of modified microbes to lower the energy intensity of mining or materials production) are likely to be quite different from attitudes toward ‘wild’ deployment (e.g. plants or microbes engineered to sequester atmospheric carbon).

Justice/distributive impacts: Beyond impacts on existing industries, wider social justice and equity concerns arise from distributional effects. One lesson of the Green Revolution was that new technologies generate public opposition if they amplify inequalities in society^20^. Comparable questions surround contemporary advances in EngBio. Critics might ask whether the early development of the bioeconomy has amplified international inequalities, and whether First Nations have been appropriately involved in and benefited from using genetic resources^21,22^. Mitigation-focused applications are motivated by the public good of avoiding dangerous climate change and may be less vulnerable to these critiques; however, applications with limited private benefits are unlikely to win political support from industry and so may be unusually vulnerable to opposition. Consequently, we speculate that societal tolerance for EngBio’s mitigation applications will be likely to reflect public support for other EngBio applications in the same sector (e.g. public attitudes toward a new agricultural application will be correlated with public acceptance for gene editing in agriculture rather than in healthcare)^23^.

Decision-making about climate-linked technologies needs to consider the following dilemma: while communities in the Global South will face the worst impacts of climate change, research capacity has primarily been located in the Global North (plus China)^3,24^. This dilemma suggests that decision-making on potentially controversial applications should ideally occur in multilateral fora^25^, and that efforts to expand research, regulation and policy capacity in the Global South are a necessary precursor to global decision-making. The Degrees Initiative, which has assisted Southern scientists to develop expertise in respect of solar geoengineering, is a useful example of an effort to internationalise expertise *prior* to global deliberation in respect of governance^26^.

### Barriers

**Fig. 1:**
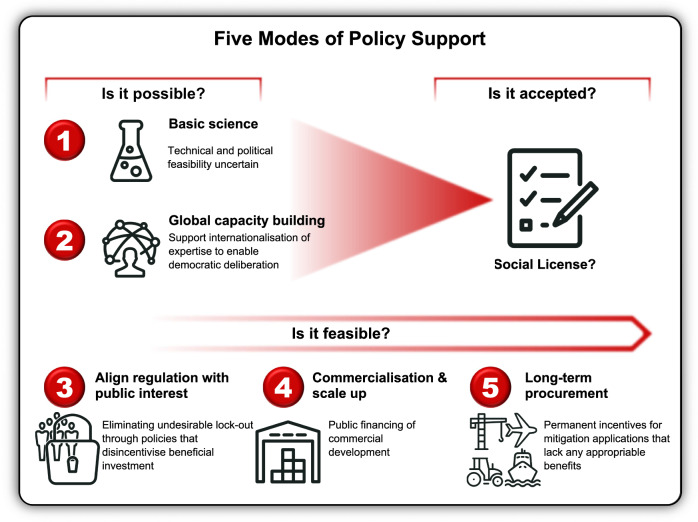
Five Modes of Policy Support. Funding for basic science (1) should ideally be supplemented by measures (2) building global capacity by internationalising expertise in, and public deliberation on, EngBio mitigation applications. A democratic assessment of public interest in EngBio applications should also allow policymakers to eliminate undesirable lock-out by (3) aligning regulation with public interest*.* In the case of technologies that have limited appropriable economic benefits, more active support for (4) commercialisation and scale-up through policies such as direct public financing, taxation credits or procurement policies may be needed. Where applications’ only purpose is to reduce greenhouse gas emissions (5) long-term procurement policies will be needed if large-scale deployment is necessary to achieve climate goals (e.g. direct air capture technologies).

Appropriability and lock-out are two attributes of technology that shape the prospects for private-sector commercialisation^24^. Table 2 shows how these drivers combine when assessing the likelihood of deploying an EngBio climate mitigation application at a commercially and environmentally relevant scale, while Fig. 1 summarises the types of policy interventions that might help to overcome barriers and ultimately support the deployment of EngBio applications at climate-relevant scales.

**Table 2:** Examples of appropriability and lock-out of EngBio climate mitigation applications

Appropriability	Lock-out risk	
	High	Low
High	Profitable applications of EngBio that are likely to face strong political resistance from established industries.	Profitable applications of EngBio that are likely to confront limited resistance from established industries.
Low	*E.g. Synthetic animal proteins threaten agricultural producers that are often politically organised*.	*E.g. Synthetic biofuels enter a liquid global market; within many importer states there are no local producers so political opposition may be ineffective*.
	EngBio applications that reduce GHG emissions but do not compete with existing industries will rarely encounter established industry opposition.	EngBio applications that reduce GHG emissions but do not compete with existing industries are unlikely to face significant established industry opposition.
	*E.g. Environmental release of GHG-consuming organisms*.	*E.g. Direct air capture in contained facilities*.

Adapted from David Victor *Global Warming Gridlock* CUP 2011*, p.130*.

*©* David G. Victor 2011. Reproduced with permission of The Licensor through PLSclear.

#### Appropriability

The term ‘appropriability’ refers to the extent to which investors can capture the value that arises if a technology proves useful. EngBio climate applications might require investments in basic science, commercialisation, and ongoing procurement policies—depending on the level of appropriable benefits they bring. All technologies that mitigate GHG emissions face an appropriability challenge: private investors are unlikely to capture the social benefits (positive externalities) that arise from reducing GHG emissions, so low-GHG technologies will generally be adopted at a sub-optimal rate. In places where robust emissions pricing schemes exist, investors can capture some of the value of supplying positive externalities. However, only about 23% of global GHG emissions are subject to a carbon price, and only a tiny proportion are priced in the $US40-80/tCO_2_eq range consistent with meeting the Paris Agreement’s temperature goals^27^. Consequently, mitigation technologies will be developed and deployed too slowly without targeted policy interventions. EngBio applications whose sole benefit is eliminating GHG emissions (e.g. a novel method of direct air capture of GHGs) face a higher appropriability challenge and are unlikely to be developed without supportive policy interventions; commercial products for which mitigation is a side-benefit can be anticipated to be developed and deployed only at a sub-optimal level.

#### Lock-out

Lock-out describes the technical, political, and regulatory barriers to market access^24^. Laws that prohibit the sale of synthetic animal proteins, require warning labels, or restrict how such products can be marketed are examples of regulatory lock-out. Analysis of lock-out overlaps with an assessment of political feasibility^18^. While it is appropriate to regulate many EngBio activities (e.g. restrictions on the environmental release of novel organisms), some forms of lock-out may be undesirable, such as when an incumbent industry uses their political and economic power to limit the market for new products. Laws restricting the use of specific terms (e.g. ‘sausage’ and ‘milk’) or the production and transport of products derived from gene technology (e.g. the Cartagena Biosafety Protocol) reflect this pattern of ‘lock-out’ of emerging industries. Innovation policy often responds to these dynamics by seeking to eliminate regulatory lock-out by incumbent industries. Regarding EngBio, policymakers should focus on aligning barriers to deployment with a democratic assessment of public interest. As noted, some applications of EngBio – e.g. environmental release of GHG-consuming microorganisms—require appropriate regulatory control, and there is a risk of ‘lone wolf’ or ‘rogue’ deployment. Governance in this sector should thus seek to enable climate-informed, democratic control over deployment decisions rather than eliminate all forms of lock-out.

#### Technological readiness

Technological readiness, which describes the level of development needed before a technology can be commercially deployed, has implications for policy support. Technological readiness determines the depth of the ‘valley of death’ between early-stage science and deployment that innovation literature describes as forestalling the development of many potential innovations^24^. Procurement policies (e.g. direct government procurement of negative emissions via reverse auctions, as currently occurs in the UK) may be sufficient to incentivise the commercialisation of technologies that require relatively little technological development. However, mitigation applications requiring significant technological development will only progress if this development process receives targeted and sustained policy support. Whether a specific EngBio climate application should be prioritised will depend on the scale of potential mitigation contribution, and the wider environmental and social impacts that will shape public acceptance and political feasibility of large-scale deployment^18^.

### Potential EngBio contributions to climate mitigation

EngBio’s possible contributions to climate mitigation are diverse. In addition to supporting many of the decarbonising strategies in the IPCC’s Integrated Assessment Models (e.g. the production of biofuels might accelerate the decarbonisation of long-distance transport), EngBio might facilitate other decarbonisation strategies that are not widely contemplated. For example, there are several routes through which engineered plants or microbes could enable direct air capture of CO_2_ or other greenhouse gases (negative emissions)^28^.

Table 3 illustrates EngBio’s potential climate mitigation contribution by mapping some potential EngBio applications against significant mitigation challenges characterised by the IPCC^29^.

**Table 3:** Select EngBio climate mitigation applications

Application Type	Examples
A: Replace fossil fuels in transport	*Biofuel from agricultural biomass or atmospheric/oceanic CO*_*2*_ Electricity storage/ rewired carbon fixation: Electromicrobial production (EMP) that combines engineered biological and electronic components might convert CO_2_ into high-density, non-volatile fuels or energy storage polymers^55,56^. Engineered *E. coli* directly uses carbon dioxide (CO_2_) to produce biofuels, bypassing photosynthesis in the production of biofuels. (e.g. Ginkgo Bioworks)^57,58^. *Hydrogen* Dark fermentation processes produce hydrogen from biomass using engineered microbial consortia^59^.
B: Reduce emissions from production processes: industry, construction and agriculture	*Industrial processes* Absorb CO_2_ waste streams and sequestration or conversion to useful by-products e.g., ethanol or sequestration^28^.
	*Construction/Materials* Cement/concrete: Biomineralisation via application of bacteria during the preparation of mortar or concrete—leading to reduced carbon emissions and longer-lasting, self-repairing concrete^60^.
	*Agriculture* N_2_OR and MMO-transformed plants capable of reducing emissions (or drawing down atmospheric) N_2_O and methane CH_4_^47,61^. Low-methane rice. Rice varieties that do not require irrigation, with altered methane/carbon fluxes, or increased yield^62^.
C: Substitutes for emissions-intensive products	*Animal proteins* Biofermentation: Synthetic animal proteins (displacing CH_4_), meat and milk^8,63^.
	*Fertilisers* Cultivation or engineering of nitrogen-fixing microbes (or plants/biofertilisation) is already at early-stage commercialisation^9^.
	*Chemical manufacturing* Building metabolic pathways in microbes for Hydrogen production which is not dependent on fossil fuel feedstock^64^.
D: Sequester or mitigate in environment	*Sequester or mitigate carbon/GHGs in environment* Engineering crop root systems for enhanced carbon sequestration Increased production of suberin—a lipophilic complex polyester biopolymer; Microbes transforming organic carbon into stable carbonates^47^. Direct air capture carbon/ non-carbon GHGs^61^.

This overview points toward four major pathways through which EngBio might contribute to mitigation, i.e. (i) replacing some *fossil fuels*^3^—(e.g. use of zero-carbon energy in production of synthetic fuels to replace jet fuel, bunker fuel, etc.); (ii) harnessing production process emissions in industry, construction and agriculture by reusing ruminant and waste emissions (e.g. capture and utilisation of gaseous carbon waste streams as biomanufacturing feedstocks); (iii) bio-based product substitution for emissions-intensive industries (e.g. production of milk proteins via precision fermentation to eliminate methane emissions; hydrogen-generating or nitrogen-fixing microbes that replace fossil fuel derived fertilisers; (iv) direct environmental sequestration *of* greenhouse gases outside of industrial systems, or storage of carbon waste via bio-based production specifically engineered for long-lived carbon deposition (e.g. engineered carbon-capturing plants, microbes and bio-based carbon waste streams). The case for environmental sequestration arises because negative emissions of 100–1000 metric gigatons of CO_2_ by 2100 are anticipated in IPCC scenarios that limit warming to 1.5 °C, and the pathway to this scale of negative emissions is not currently clear^30^. The authors assume these four application areas would be scaled with other well-characterised carbon-neutral or negative energy sources (e.g. solar, geothermal, wind and nuclear) to achieve climate mitigation objectives.

#### Replacing fossil fuels

Applying synthesis to the production of biofuels would be EngBio’s fastest and least disruptive path to gigatonne-scale mitigation. Production of carbon-neutral fuels could eliminate emissions without the slow and carbon-costly process of replacing existing infrastructure with new technologies (vehicles, planes, power stations, etc.). However, in addition to scaling challenges, producing sustainable biofuels would require resolving other difficulties. The climate and environmental impacts of synthesised biofuels reflect both the carbon footprint of their total energy inputs (e.g. the carbon intensity of electricity) and the original source of their embodied carbon and its land footprint (e.g. a biogenic source versus carbon captured from the combustion of fossil fuels). Debates over the energy return on investment and environmental impacts of corn-based ethanol production reflect how dependent the environmental impacts of biofuels are on their inputs^12,13^.

Methods that produce biofuels from waste phytomass (e.g. organic waste from agriculture and cities) rather than valuable commodities (e.g. glucose from corn) could radically reduce biofuel production’s environmental and social impacts. Waste feedstocks are economically promising due to their abundance and low cost, and they can often be locally sourced from city waste^31^. Most significantly, using low-value feedstocks would mean that biofuels and food production were no longer in competition. However, greater technical challenges exist in producing fuel from heterogeneous waste products instead of higher-value, controlled inputs (e.g. glucose), as seen in work to refactor organisms to fix biomass from carbon^32,33^. Another key advantage of biofuels is that they may be highly compatible with a future energy system dominated by intermittent renewables. Renewable-dominated grids require redundancy at some point in the energy system, as peaks and lows in wind and solar generation do not align with demand. Technologies with high capital costs (e.g. alkaline electrolysis of hydrogen) are not ideally suited to solving the redundancy problem due to high utilisation rate requirements for profitability. If EngBio enables production methods with relatively low capital costs that are profitable with low utilisation rates, then EngBio solutions could fulfil the redundancy role in renewables-dominated grids.

##### Production process mitigation

Capture and reuse of gaseous carbon waste streams is another promising application of EngBio. These carbon waste streams could be sourced from heavy industry and used as feedstocks to synthesise products such as protein or next-generation biofuels^34^. However, many technical challenges would need to be overcome, such as separating or tolerating heavy metals and toxins in industrial carbon waste streams and achieving cost parity with conventional chemical products and oil-based supply chains. Some opponents express concerns that developing profitable uses for carbon waste streams will create a moral hazard if it supports the continued operation of carbon-intensive industrial applications like coal-fired power generation^35^. An alternative perspective notes that the oil and gas sectors possess sufficient capital and expertise to either thwart effective climate mitigation policies, or to scale gigatonne atmospheric carbon capture if policy adequately incentivises industrial transformation. One development path for chemical synthesis from gaseous carbon waste streams that is being pursued by firms such as LanzaTech would see initial deployments focus on the chemical synthesis of high-value chemical compounds like vanillin, acetone or isopropanol^36^. However, such shifts to chemical production must be assessed on a case-by-case basis due to their potential for negative environmental consequences^37^. Over time, if this technology were to become ubiquitous, the associated scale effects may decrease the cost structure of biofuel synthesis towards cost parity with conventional petrochemicals. In addition to carbon capture and utilisation, EngBio might play a role in carbon capture and sequestration/deposition strategies within industrial systems. For example, bacteria could be used in CO_2_ fixation (either in a liquid of solid form) at industrial carbon capture facilities that treat exhaust gas and other waste streams in a two-product strategy^38^. The primary product would constitute the high-value synthesis target, and the secondary product would be a carbon-dense by-product (such as biochar) optimised as a fermentation waste stream. This carbon-dense waste stream could then be diverted into long-term deposition or sequestration for an economic return dependent on carbon pricing and subsidy policies.

##### Product substitution

The use of engineered chemical synthesis to produce substitutes for carbon-intensive products could become a widespread climate mitigation technique. Biosynthesis (or biomanufacturing) is a production route whereby a biological device (typically based on a single-celled chassis organism like yeast or bacteria) is designed to transform a chemical input into a high-value complex chemical output. Product substitution through synthesis has widespread theoretical potential across chemical and energy sectors. Contemporary biofoundries (genome foundries) have industrialised the biodesign process for these biological devices. Early commercial applications of EngBio have focused on synthesising very high-value chemical outputs where small-scale production is profitable^39^, such as the pharmaceutical supply chain work undertaken by Antheia. Applying synthesis to the commercial production of bulk commodities would require overcoming various challenges associated with scaling biological processes^40,41^, aptly demonstrated by LanzaTech^42^. If EngBio is to provide mitigation on a climate-relevant scale (i.e. gigatonnes of carbon equivalent), such scaling challenges will need to be resolved either within contained production facilities, or outside of them. Significant investment in gigatonne-scale industrial fermentation facilities optimised for gaseous carbon waste reuse and atmospheric capture is required to mitigate worst-case climate change. However, less investment may be required for solutions deployed into nature that will scale their biomass and carbon deposition strategies through organic processes, for example, engineered cyanobacteria released into the ocean with enhanced carbon deposition layering the ocean floor. The uncontrolled-release of such solutions may not be possible due to public opposition linked to anticipated biosecurity and environmental impacts.

Precision fermentation is already being used to develop synthetic substitutions for palm oil (e.g. by C16 Biosciences). Widespread utilisation of synthetic palm oil would likely have a tangible impact on forest clearing practices, a major source of GHG emissions^38^. However, biomanufactured palm oil would negatively impact those lower-income economies that rely on palm oil exports^43^. Other agricultural applications include synthetic alternatives for animal products, including milk and animal fats. Synthetic alternatives for animal and aquaculture feed are also being developed and may reduce agricultural GHG emissions. However, as the example of synthetic palm oil demonstrates, there will likely be some negative economic and social consequences, including disruption in countries that rely on agricultural exports. Such disruption may include job losses, industrial decline and, in some cases, the collapse of entire economic sectors. Thus, policy interventions should be sensitive to negative upstream and downstream effects. It also remains to be seen whether, and to what extent, alternative EngBio applications offer viable, long-term solutions and do not inadvertently contribute to the environmental challenges they are designed to resolve^44^.

##### Direct sequestration

developing applications that operate outside contained production facilities might be another path to large-scale carbon capture. Examples include engineered plants for agricultural applications^45,46^, or—more controversially—environmental release of engineered microbes that consume carbon or methane^47^. The risks associated with the environmental release of novel organisms would require scrutiny and, even without identified risks, could be anticipated to encounter public opposition^23^. Since interventions interfacing engineered organisms with ecosystem components involve high risks (e.g. ecosystem damage) and high potential rewards (e.g. avoided climate harms), they raise significant questions about how democratic deliberation should inform policy. Public attitudes will also play a determinative role in the success of many applications that interface with human cultures^48^. Policy mechanisms that prioritise the development of specific applications of EngBio and—potentially—regulate and limit the development of others will need to be responsive to political feasibility and public opinion.

## A way forward

EngBio is an exciting tool with enormous promise in supporting efforts to achieve net zero emissions^49^, alongside wider economic and social benefits^50^. This article has outlined four major pathways through which EngBio could contribute to climate mitigation (Table 3): replacement of fossil fuels; elimination of emissions from production processes; substitution of carbon-intensive products; and, the direct sequestration of carbon dioxide and other greenhouse gases within, and outside of, industrial systems. We have argued that policy development is needed to support promising EngBio mitigation applications through development and, where needed, long-term deployment. Figure 1 summarises the types of global and national policy interventions that will further the goals of building scientific capacity in the global south; aligning national regulation with public interest; and, supporting commercialisation with public financing, taxation credits or procurement policies.

At this early stage in the development of EngBio mitigation applications, it is too early to predict which technological pathways will have the greatest technical potential and political feasibility^51^. The fine-grained work of identifying the most promising applications, supporting their technological development, eliminating regulatory barriers, and crafting incentives for deployment will necessarily occur within national political communities and policy-making bodies. However, there are some specific near-term enabling steps that the global climate and EngBio policy communities should take to support this work. We identify three specific steps that would raise the profile of engineering biology within climate policy bodies and build global capacity to ensure that EngBio development addresses the needs of communities in the Global South:

i. the IPCC’s *Working Group III on Mitigation of Climate Change* should conduct a comprehensive assessment of EngBio’s mitigation applications since the IPCC has the capacity to promote both international understanding of the sector’s potential and more active deliberation on desirable futures^52^.
ii. climate funding agencies (Green Climate Fund, public sector donors, etc.) should support the development of engineering biology research capacity across the global south. Some funding might be directed to organisations such as CGIAR (formerly the Consultative Group for International Agricultural Research—a network of not-for-profit, agricultural R&D agencies), which have established reputations for generating public goods in low-income countries^46^.
iii. existing global initiatives promoting cooperation in respect of engineering biology, (e.g. the Global Future Council on the Future of Synthetic Biology, EBRC Global Forum) should work to develop climate mitigation goals and model policies to incentivise the development of climate-relevant EngBio applications. This effort would be analogous to the governance work done in respect of risk^53^. One goal should be to ensure that EngBio is routinely integrated into existing and new low-carbon innovation policies (e.g. policies that support commercialisation and scale-up of non-appropriable technologies through direct public financing, taxation credits, long-term procurement policies such as reverse auctions, and policies to promote transparency and public engagement)^54^.

There remains a vast gap between the mitigation potential of EngBio and its realisation. Translating research into suitable policy frameworks is central to actualising EngBio applications at gigatonnes-of-mitigation-scale.

## References

[1] Riahi, K. et al. in *Climate Change 2022: Mitigation of Climate Change. Contribution of Working Group III to the Sixth Assessment Report of the Intergovernmental Panel on Climate Change* (eds Shukla, P. R. et al.) Ch.3 (Cambridge Univ. Press, 2022).

[2] Engineering Biology Research Consortium. *Engineering Biology: A Research Roadmap for the Next-Generation Bioeconomy*. http://roadmap.ebrc.org (2019).

[3] National Academies of Sciences, Engineering, and Medicine. *Safeguarding the Bioeconomy* (National Academies Press, 2020). 32352690

[4] Kemp L (2020). Point of view: bioengineering horizon scan 2020. eLife.

[5] The White House Office of Science and Technology Policy. Bold goals for U.S. Biotechnology and Biomanufacturing. https://www.whitehouse.gov/wp-content/uploads/2023/03/Bold-Goals-for-U.S.-Biotechnology-and-Biomanufacturing-Harnessing-Research-and-Development-To-Further-Societal-Goals-FINAL.pdf (2023).

[6] UK Department for Science, Innovation& Technology. National vision for engineering biology*.* https://www.gov.uk/government/publications/national-vision-for-engineering-biology/national-vision-for-engineering-biology#executive-summary (2023).

[7] Tan X, Letendre JH, Collins JJ, Wong WW (2021). Synthetic biology in the clinic: engineering vaccines, diagnostics, and therapeutics. Cell.

[8] Graham AE, Ledesma-Amaro R (2023). The microbial food revolution. Nat. Commun..

[9] Davis WG, Bonini Pires CA, Ruiz Diaz DA, Roozeboom K, Rice CW (2020). Pivot bio proven inoculant as a source of nitrogen in corn. Kans. Agric. Exp. Station Res. Rep..

[10] Aklin, M. & Urpelainen J. *Renewables: The Politics of a Global Energy Transition* (MIT Press, 2018).

[11] Gauvreau D, Winickoff D, Philp J (2018). Engineering biology and the grand challenges: do we need a new R&D&I model?. Eng. Biol..

[12] ARAP-E. ECOSynBio program*:* energy and carbon optimized synthesis for the bioeconomy. https://arpa-e.energy.gov/technologies/programs/ecosynbio (2021).

[13] Mission Innovation. Innovation challenge 5: converting sunlight into solar fuels and chemicals roadmap 2020–2050. http://mission-innovation.net/wp-content/uploads/2021/03/Converting-Sunlight-into-Solar-Fuels-and-Chemicals-MI-Challenge-5-roadmap-Feb-2021-final.pdf. (2021)

[14] Mission Innovation. Launch of the integrated biorefineries mission press release. https://mission-innovation.net/2022/04/04/4th-april-2022-launch-of-the-integrated-biorefineries-mission-press-release/ (2022).

[15] President Biden. Executive order 14081, advancing biotechnology and biomanufacturing innovation for a sustainable, safe, and secure American bioeconomy. https://www.federalregister.gov/documents/2023/04/27/2023-08841/executive-order-14081-advancing-biotechnology-and-biomanufacturing-innovation-for-a-sustainable-safe (2022).

[16] Department of Energy. DOE launches new office to coordinate critical and emerging technology. https://www.energy.gov/articles/doe-launches-new-office-coordinate-critical-and-emerging-technology (2023).

[17] Cheng F, Luo H, Jenkins JD, Larson ED (2023). Impacts of the inflation reduction act on the economics of clean hydrogen and synthetic liquid fuels. Environ. Sci. Technol..

[18] Jewell J, Cherp A (2020). On the political feasibility of climate change mitigation pathways: is it too late to keep warming below 1.5°C?. Wiley Interdiscip. Rev. Clim. Change.

[19] Hobman E, Mankad A, Carter L (2022). Public perceptions of synthetic biology solutions for environmental problems. Front. Environ. Sci..

[20] Pingali PL (2012). Green revolution: impacts, limits, and the path ahead. Proc. Natl Acad. Sci. USA.

[21] Suiseeya KRM (2014). Negotiating the Nagoya protocol: indigenous demands for justice. Glob. Environ. Politics.

[22] Stoianoff, N. In *Sustainability and Law: General and Specific Aspects* (eds Rupo, D. et al.) Ch. 22 (Springer International Publishing, 2020).

[23] Herring R, Paarlberg R (2016). The political economy of biotechnology. Annu. Rev. Resour. Econ..

[24] Victor, D. G. *Global Warming Gridlock: Creating More Effective Strategies for Protecting the Planet* (Cambridge Univ. Press, 2011.).

[25] Dixon TA, Freemont PS, Johnson RA, Pretorius IS (2022). A global forum on synthetic biology: the need for international engagement. Nat. Commun..

[26] Visioni D (2023). The scientific and community-building roles of the Geoengineering Model Intercomparison Project (GeoMIP)-past, present, and future. Atmos. Chem. Phys..

[27] World Bank. *State and Trends of Carbon Pricing 2023* (World Bank, 2023).

[28] Onyeaka H, Ekwebelem OC (2023). A review of recent advances in engineering bacteria for enhanced CO_2_ capture and utilization. Int. J. Environ. Sci. Technol..

[29] Dhakal, S. et al. in *Climate Change 2022: Mitigation of Climate Change. Contribution of Working Group III to the Sixth Assessment Report of the Intergovernmental Panel on Climate Change* (eds. Shukla, P. R. et al.) Ch. 2 (Cambridge Univ. Press 2022).

[30] Maher B, Symons J (2022). The international politics of carbon dioxide removal: pathways to cooperative global governance. Glob. Environ. Politics.

[31] National Academies of Sciences, *Engineering, and Medicine. Gaseous Carbon Waste Streams Utilization: Status and Research Needs* (The National Academies Press, 2019).

[32] Gleizer S (2019). Conversion of *Escherichia coli* to generate all biomass carbon from CO_2_. Cell.

[33] Gassler T (2020). The industrial yeast *Pichia pastoris* is converted from a heterotroph into an autotroph capable of growth on CO2. Nat. Biotechnol..

[34] Wilson IAG, Styring P (2017). Why synthetic fuels are necessary in future energy systems. Front. Energy Res..

[35] Lutzke L, Árvai J (2021). Consumer acceptance of products from carbon capture and utilization. Clim. Change.

[36] Liew FE (2022). Carbon-negative production of acetone and isopropanol by gas fermentation at industrial pilot scale. Nat. Biotechnol..

[37] Dalziell J, Rogers W (2022). Are the ethics of synthetic biology fit for purpose? A case study of artemisinin. Proc. IEEE.

[38] French KE (2019). Harnessing synthetic biology for sustainable development. Nat. Sustain..

[39] Wiltschi B (2020). Enzymes revolutionize the bioproduction of value-added compounds: from enzyme discovery to special applications. Biotechnol. Adv..

[40] Mota GF (2022). Biodiesel production from microalgae using lipase-based catalysts: current challenges and prospects. Algal Res..

[41] Ingelman, H. et al. Autotrophic adaptive laboratory evolution of the acetogen Clostridium autoethanogenum delivers the gas-fermenting strain LAbrini with superior growth, products, and robustness. Preprint at *bioRxiv*10.1101/2023.01.28.526018 (2023). 10.1016/j.nbt.2024.06.002 38871051

[42] Köpke M (2022). Redesigning CO_2_ fixation. Nat. Synth..

[43] Meijaard E (2020). The environmental impacts of palm oil in context. Nat. Plants.

[44] Buck HJ (2020). Evaluating the efficacy and equity of environmental stopgap measures. Nat. Sustain..

[45] DeLisi C (2019). The role of synthetic biology in climate change mitigation. Biol. Direct.

[46] Pixley (2019). Genome editing, gene drives, and synthetic biology: will they contribute to disease-resistant crops, and who will benefit?. Annu. Rev. Phytopathol..

[47] Schweitzer H (2021). Innovating carbon-capture biotechnologies through ecosystem-inspired solutions. One Earth.

[48] Carter L, Mankad A, Hobman EV (2023). Is public engagement in bioengineering and synthetic biology improving research outcomes?. OMICS.

[49] Fulvi, D. & Wodak, J. Using synthetic biology to avert runaway climate change: a consequentialist appraisal. *Ethics Policy Environ.* **1**, 89–107 (2023).

[50] McGregor A (2021). Just food transitions? The social benefits of alternative proteins. Food Aust..

[51] Sloan WT, Gómez-Borraz TL (2023). Engineering biology in the face of uncertainty. Interface Focus.

[52] Livingston JE, Rummukainen M (2020). Taking science by surprise: the knowledge politics of the IPCC Special Report on 1.5 degrees. Environ. Sci. Policy.

[53] Undheim TA (2024). The whack-a-mole governance challenge for AI-enabled synthetic biology: literature review and emerging frameworks. Front. Bioeng. Biotechnol..

[54] Honegger M, Poralla M, Michaelowa A, Ahonen HM (2021). Who is paying for carbon dioxide removal? Designing policy instruments for mobilizing negative emissions technologies. Front. Clim..

[55] Salimijazi F, Parra E, Barstow B (2019). Electrical energy storage with engineered biological systems. J. Biol. Eng..

[56] Salimijazi F (2020). Constraints on the efficiency of engineered electromicrobial production. Joule.

[57] Rodriguez K (2023). Gas fermentation for microbial sustainable aviation fuel production. Microbiol. Aust..

[58] Lu H (2022). Bioengineered microbial platforms for biomass-derived biofuel production–A review. Chemosphere.

[59] Wang S, Zhang T, Bao M, Su H, Xu P (2020). Microbial production of hydrogen by mixed culture technologies: a review. Biotechnol. J..

[60] Kadapure SA (2021). The biotechnology approach for sustainable concrete material – a review. Mag. Concr. Res..

[61] Strand SE, Zhang L, Flury M (2022). Theoretical analysis of engineered plants for control of atmospheric nitrous oxide and methane by modification of the mitochondrial proteome. ACS Sustain. Chem. Eng..

[62] Du L (2021). Comprehensive analysis of SUSIBA2 rice: the low-methane trait and associated changes in soil carbon and microbial communities. Sci. Total Environ..

[63] Post MJ (2020). Scientific, sustainability and regulatory challenges of cultured meat. Nat. Food.

[64] Willows, R. et al. Recombinant microorganisms and process. Patent 2021246542 (2021).

